# Xanthogranulomatous pyelonephritis presenting as acute pleuritic chest pain: a case report

**DOI:** 10.1186/s13256-017-1277-4

**Published:** 2017-04-12

**Authors:** Justin Chow, Rameez Kabani, Kirstie Lithgow, Magdalena A. Sarna

**Affiliations:** 1 Department of Medicine, University of Calgary, Calgary, Alberta Canada; 2 Department of Medicine, Division of Endocrinology and Metabolism, University of Calgary, Calgary, Alberta Canada

**Keywords:** Xanthogranulomatous pyelonephritis, Pyelonephritis, Pleural effusion, Pleuritic chest pain, Pleurisy

## Abstract

**Background:**

Xanthogranulomatous pyelonephritis is a rare and serious manifestation of chronic kidney inflammation that can be life-threatening if not recognized and treated appropriately, often with antibiotics and surgery. Affected patients are most commonly females in their fifth or sixth decade of life with a background of obstructive uropathy, nephrolithiasis, or recurrent urinary tract infections who present with vague nonspecific symptoms.

**Case presentation:**

A 43-year-old woman of Russian ethnicity with a history of nephrolithiasis presented to our emergency department with new left-sided pleuritic chest pain amid a 6-week history of constitutional symptoms including fevers, night sweats, and 7 kg of weight loss. Workup for acute coronary syndrome and pulmonary embolism in our emergency department was negative. Given that she was clinically unwell, she was admitted to internal medicine to expedite workup for the cause of her symptoms. A broad differential diagnosis for various infectious, inflammatory/autoimmune, and neoplastic processes was considered. Based on classic radiographic and histopathologic findings, she was ultimately diagnosed with xanthogranulomatous pyelonephritis of her left kidney, which was a direct consequence of chronic inflammation. This inflammation exhibited spread to local tissues and across her left hemidiaphragm, resulting in a unilateral pleural effusion which explained her chest discomfort. She was treated with antibiotics administered intravenously and urgent total nephrectomy with a good functional outcome.

**Conclusions:**

Our case illustrates an uncommon but clinically important do-not-miss diagnosis that underlies a common clinical presentation of pleuritic chest pain. The case underscores the importance of maintaining a broad differential diagnosis and organized approach when treating patients with undifferentiated clinical presentations.

## Background

Xanthogranulomatous pyelonephritis (XGP) is a rare manifestation of chronic kidney inflammation that can be life-threatening if not recognized early and treated appropriately. The prototypical patient affected by this condition is a female in her fifth or sixth decade of life with a background of obstructive uropathy presenting with nonspecific constitutional symptoms [1]. Diagnosis of XGP is usually made radiographically and later confirmed by histopathology. Treatment most often involves a combination of surgery and antibiotics [1, 2]. Here, we describe a very atypical case of XGP in which the patient’s presenting symptom was acute-onset pleuritic chest pain.

## Case presentation

A 43-year-old woman of Russian ethnicity presented to our emergency department with approximately 24 hours’ duration of subxiphoid pleuritic chest pain radiating to her left shoulder tip and flank. Her past medical history was significant for nephrolithiasis (she did not recall stone type but reported to be managed conservatively) and obesity. She also indicated that she had intermittent fevers, night sweats, and a 7 kg weight loss over the previous 6 weeks. She denied any urological symptoms such as hematuria, dysuria, increased frequency, or urgency. She denied any recent travel or family history of autoimmune or malignant diseases. She had no history of diabetes mellitus or immune suppression. She was a lifetime non-tobacco smoker and consumed alcohol only on rare occasions. On physical examination, her vital signs were significant for the following: temperature 36.2 °C, blood pressure of 138/96 mmHg (sitting), heart rate of 130 beats per minute (regular), and respiratory rate of 22 breaths per minute. Pulse oximetry showed an oxygen saturation of 97% on room air. She was felt to be intravascularly volume deplete on the basis of dry mucous membranes, depressed jugular venous pressure, and resting tachycardia. A thorough examination did not localize any potential sources of infection, nor was there a palpable abdominal mass. Her initial investigations (shown in Table 1) revealed a peripheral white blood cell (WBC) count of 12.9×10^9^ (with predominant neutrophilia), elevated C-reactive protein of 17.9 mg/dL, and new microcytic anemia with hemoglobin of 88 g/L. A review of her prior laboratory work revealed that her hemoglobin had previously been normal at 130 g/L 2 years prior, but her thrombocytosis (with platelets between 400 and 600×10^9^) had been present since 2012. No other abnormalities in terms of serum chemistry and renal function were shown on previous laboratory work. Her urine analysis contained WBCs but the final urine culture was negative. There were no red blood cells in the urine. Further review of her past urine studies revealed persistent sterile pyuria dating at least back to 2009. A limited workup for acute coronary syndrome – including serial 12-lead electrocardiograms and serum troponins – was negative. A chest computed tomography (CT) scan for suspected pulmonary embolism was performed which showed a small left-sided pleural effusion as well as a loculated effusion measuring 10.3×3.4×5.6 cm in the subcapsular region surrounding her spleen.

**Table 1:** Admission bloodwork

Test	Result	Reference range
CBC	Hb 88 g/L	120–160 g/L
	WBC 15.8×10^9^	2.0–9.0×10^9^
	Plt 559×10^9^	150–400×10^9^
	MCV 70 fL	82–100 fL
Electrolytes	Na 133 mmol/L	133–145 mmol/L
	K 3.9 mmol/L	3.3–5.1 mmol/L
	Cl 98 mmol/L	98–111 mmol/L
	HCO_3_^−^ 22 mmol/L	21–31 mmol/L
	Urea 2.9 mmol/L	2.0–7.0 mmol/L
	Calcium 2.12 mmol/L	2.10–2.55 mmol/L
	Phosphate 1.10 mmol/L	0.80–1.50 mmol/L
	Magnesium 0.88 mmol/L	0.65–1.05 mmol/L
Creatinine	72 umol/L	35–100 umol/L
Random glucose	8.2 mmol/L	3.3–11.0 mmol/L
Endocrine	HbA1c 6.3%	4.3–6.1%
	TSH 3.41 mIU/L	0.20–4.00 mIU/L
Liver function panel	LD 141 U/L	8–35 u/L
	ALT 22 U/L	1–40 U/L
	ALP 207 U/L	30–115 U/L
	GGT 110 U/L	8–35 U/L
	Total bilirubin 7 umol/L	0–24 umol/L
	Lipase 16 U/L	0–80 U/L
	INR 1.4	0.9–1.1
C-reactive protein	17.9 mg/dL	0.0–8.0 mg/dL
Erythrocyte sedimentation rate	107 mm/hour	0–20 mm/hour
D-dimer	1.81	<0.46 FEU
Troponin T (high-sensitivity)	<3 ng/L	0–14 ng/L

*ALP* alkaline phosphatase, *ALT* alanine aminotransferase, *CBC* complete blood count, *Cl* chlorine, *GGT* gamma-glutamyltransferase, *Hb* hemoglobin, *HbA1C* glycated hemoglobin, *HCO*_*3*_^*−*^ bicarbonate, *INR* international normalized ratio, *K* potassium, *LD* lactate dehydrogenase, *MCV* mean corpuscular volume, *Na* sodium, *Plt* platelets, *TSH* thyroid-stimulating hormone, *WBC* white blood cell

**Fig. 1:**
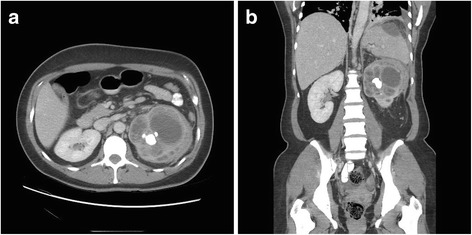
Computed tomography of the abdomen and pelvis demonstrating xanthogranulomatous pyelonephritis of the left kidney. **a** Axial section at the level of the renal calyces demonstrating markedly abnormal appearance of the left kidney. The renal pelvis is contracted around a staghorn calculus with surrounding distension and ballooning in the upper pole calyces, with surrounding soft tissue stranding and enlarged perinephric and retroperitoneal lymph nodes which are presumed to be reactive. **b** Coronal section of the abdomen and pelvis showing aforementioned changes in the left kidney with superior extension into the spleen parenchyma. Tracking of the inflammatory perinephric soft tissue along the posterior aspect of the spleen (not shown in this view) connects with a large hypodense collection under the splenic capsule, which also appears to communicate across left hemidiaphragm with resulting left-sided pleural effusion

Given her unusual clinical presentation, she was admitted to internal medicine for further investigation. A broad differential diagnosis including various infectious, inflammatory/autoimmune, and neoplastic etiologies was considered. Blood and urine cultures were sent as well as autoimmune markers including anti-nuclear antibody, serum complement levels, and rheumatoid factor. These investigations were all negative. Further imaging was obtained to look for occult sources of infection or malignancy. A dedicated CT of her abdomen and pelvis revealed a staghorn calculus in her left kidney with surrounding inflammatory soft tissue changes including dilated renal calyces overlying a paradoxically contracted renal pelvis (the classic “bear’s paw sign” of XGP [3]). These inflammatory changes extended to the aforementioned subcapsular effusion, the inferior tip of her spleen parenchyma, and also appeared to communicate across her left hemidiaphragm (see Fig. 1). Based on this constellation of imaging findings, a diagnosis of XGP was made.

She was empirically started on intravenously administered broad-spectrum antibiotics with piperacillin-tazobactam 3.375 g intravenously administered every 6 hours and urgent urological consultation was obtained. She ultimately underwent a successful laparoscopic total left nephrectomy. No definitive microbiologic diagnosis was ever made on cultures of urine, blood, and surgical specimens, even after incubation for the maximum time period of 24 hours, 5 days, and 4 weeks, respectively. Empiric piperacillin-tazobactam was continued for 7 days preoperatively and 7 days postoperatively and a decision was made to discharge the patient home on an additional 7-day course of orally administered cefixime 200 mg daily. She has been medically and functionally well for nearly 8 months after hospitalization.

**Fig. 2:**
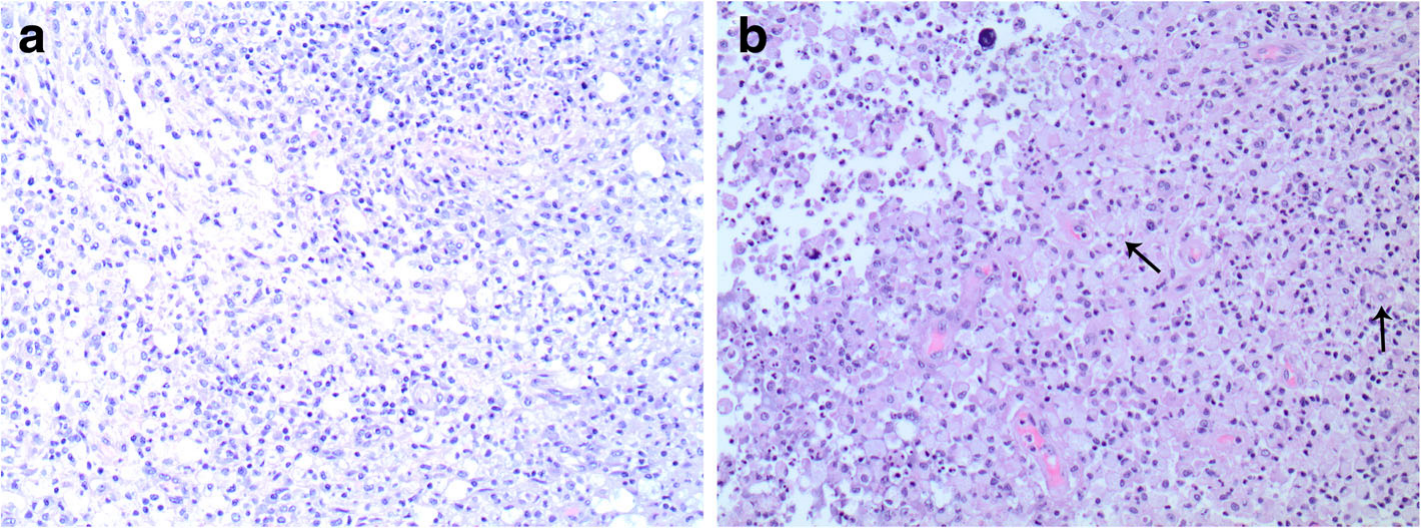
Xanthogranulomatous pyelonephritis and coexisting malakoplakia of the left kidney. Histopathological examination of the surgical specimen with hematoxylin and eosin staining reveals microscopic features of chronic inflammation. **a** Heavy lymphoplasmacytic infiltration. **b** Inflammatory infiltrates with layered sheets of histiocytes. Michaelis–Gutmann bodies pathognomonic for malakoplakia (granulomatous inflammation of the genitourinary tract) are indicated by *arrows*

Gross surgical pathology demonstrated marked abnormalities including dilated renal calyces, greenish/tan and pink purulent material, and multiple intraluminal calculi. As previously mentioned, no organisms were seen on microscopic examination, even after 4 weeks of tissue cultures. Portions of the renal capsule also exhibited deep congestion. Surrounding were four enlarged lymph nodes. On microscopic examination, these enlarged lymph nodes were histopathologically normal with no evidence of malignancy; thus they were felt to be reactive to an underlying inflammatory process. Microscopic examination of the surgical specimen (shown in Fig. 2) revealed evidence of chronic inflammation including heavy inflammatory lymphoplasmacytic infiltrates with layered sheets of histiocytes. Michaelis–Gutmann bodies – pathognomonic for malakoplakia [4] (granulomatous inflammation of the genitourinary tract heralded by the presence of intracellular inclusion bodies) – were also present. Together, these histopathological findings and previous imaging results confirmed the diagnosis of XGP with coexisting malakoplakia.

## Discussion

XGP is a chronic and severe retroperitoneal infectious/inflammatory process that manifests as chronic destructive granulomatous inflammation of renal parenchyma often resulting in complete loss of function in the affected kidney. This inflammation may invade and spread to adjacent structures, most commonly the gastrointestinal tract, urinary tract, and skin [2]. As previously mentioned, patients are typically females in their fifth or sixth decade of life; patients often present with flank pain, lower urinary tract symptoms, as well as constitutional symptoms such as recurrent fevers and weight loss [1]. Although the pathogenesis of XGP is not known, almost all cases occur in the setting of obstructive uropathy, nephrolithiasis, and/or recurrent urinary tract infections [1]. Diagnosis of XGP requires a combination of typical clinical symptoms and classic imaging features such as the loss of normal kidney contour and dilated renal calyces surrounding a paradoxically contracted renal pelvis (“bear’s paw sign” [3]) in patients with a history of urinary tract disease. Definitive diagnosis requires histopathological examination of specimens. Only recently has the coexistence of malakoplakia been reported in rare cases, suggesting that XGP and malakoplakia may in fact be two entities on the same spectrum of disease [5, 6].

Once a diagnosis of XGP is established, treatment involves a combination of systemic broad-spectrum antibiotics as well as surgical nephrectomy (partial versus total depending on extent of kidney involvement) for source control [7, 8]. Given the rarity of XGP, there are no guidelines available to inform optimal antimicrobial selection, duration, and timing of surgery; these decisions are often made at the discretion of the attending physician. Empiric broad-spectrum antibiotics (which should cover for *Staphylococcus aureus* and *Pseudomonas aeruginosa*) are continued until culture data are available to guide an appropriate stepdown regimen. If adequate source control is achieved, antibiotics are typically continued for 1 to 2 weeks postoperatively [9, 10]. As shown in two relatively large case series of 41 and 26 patients, offending pathogens are usually found by culturing resected tissues, with the two most common culprit organisms being *Escherichia coli* and *Proteus mirabilis* [10, 11]. In exceedingly rare instances of focal disease (defined as less than 10% kidney involvement [2]), XGP has been successfully treated non-surgically with prolonged courses of antibiotics and followed with serial ultrasonography. This was demonstrated in a case of a 73-year-old woman with leukemia who was treated for XGP with 2 months of intravenously administered antibiotics due to being at high surgical risk [12]. Other than in patients who are poor surgical candidates, this approach may also have applicability to renal transplant recipients with focal disease for the purposes of allograft salvage.

## Conclusions

To the best of our knowledge, this is the first reported case of a patient with XGP presenting with pleuritic chest pain as a chief symptom. Her pain was explained by local spread to and inflammatory involvement of her left hemidiaphragm, in fact an atypical presentation of this disease. Nonetheless, this case reaffirms the need to consider a broad differential diagnosis in the medically undifferentiated patient; in patients with a history of lower urinary tract pathology presenting with abdominal, flank, or even chest pain amid constitutional symptoms, it may be reasonable to consider retroperitoneal infectious/inflammatory processes such as XGP in the differential diagnosis and, in the correct clinical context, adopt a low threshold to order imaging studies of the abdomen/pelvis and obtain early urological consultation.

## Abbreviations

CT: Computed tomography
WBC: White blood cell
XGP: Xanthogranulomatous pyelonephritis
